# Association between quantitative cervical cord compression metrics and upper extremity impairments in degenerative cervical myelopathy: a cross-sectional study

**DOI:** 10.3389/fneur.2026.1728273

**Published:** 2026-02-20

**Authors:** Zhihao Wang, Changhua Lin, Xu Si, Zhengsheng Wu

**Affiliations:** 1 Department of Pathology, School of Basic Medical Sciences, Anhui Medical University, Hefei, China; 2 Department of Pathology, The First Affiliated Hospital of Anhui Medical University, Hefei, China; 3 Department of Nuclear Medicine, Mindong Hospital, Ningde, China

**Keywords:** degenerative cervical myelopathy, magnetic resonance imaging, mJOA, motor impairment, spinal cord compression

## Abstract

**Background:**

Degenerative cervical myelopathy (DCM) can lead to series of neurological dysfunction. This study aims to investigate the relationship between the compressed cervical cord and the severity of upper extremity impairments in DCM patients.

**Methods:**

47 single-level DCM patients were included from January 2023 to May 2025. Cross-sectional area (CSA), anterior-posterior width (APW), right-left width (RLW), and compression ratio (CR = APW/RLW) of the most compressed cervical cord were measured. The modified Japanese Orthopedic Association (mJOA) scale, visual analog scale (VAS), neck disability index (NDI) and the Japanese Orthopedic Association Cervical Myelopathy Evaluation Questionnaire (JOACMEQ) were used to assess upper extremity impairments. Patients were categorized into two groups based on the presence of upper extremity motor dysfunction of mJOA score. Correlation analysis was used to determine the associations between the characteristics of the cervical cord compression and upper extremity impairments. Receiver operating characteristic (ROC) curve analysis was conducted to identify critical values.

**Results:**

In patients with single-level DCM, CSA, APW, and CR of the compressed cervical cord were correlated with upper extremity outcomes, including the mJOA subscore for upper extremity motor function (ρ = 0.54, 0.54, and 0.48; *P* < 0.05), VAS score for upper extremity (ρ = −0.32, −0.44, and −0.46; *P* < 0.05), and JOACMEQ subsection for upper extremity function (ρ = 0.33, 0.30, and 0.33; *P* < 0.05). ROC analysis identified critical cutoff values (CSA < 52.6 mm^2^, APW < 4.5 mm, and CR < 31.4%) that effectively discriminate upper extremity motor function in DCM patients.

**Conclusion:**

In DCM patients, the imaging parameters of the compressed cervical cord are related to upper extremity deficits, suggesting that these characteristics could serve as potential biomarkers in diagnostic assessments and treatment planning.

## Introduction

Degenerative cervical myelopathy (DCM) is the most common cause of spinal cord impairment in adults worldwide (1, 2). A series of age-related pathologies including degeneration of ligaments, intervertebral discs and osseous tissues, can lead to chronic spinal cord compression and neurological disability (3–6). Symptoms include neck pain, motor and sensory dysfunction, dizziness, blurred vision, gastrointestinal discomfort, and hypomnesia (7, 8). Successful treatment of these symptoms has been achieved after conservative non-invasive and surgical spinal treatments (9).

The accurate and personalized diagnosis and treatment of DCM are crucial to avoid irreversible neurological deficits (10). The diagnosis and treatment of DCM require the concordance of MRI imaging findings, myelopathy symptoms, and clinical signs (11). The application of MRI can effectively characterize the structural anatomical source and the severity of cervical cord compression in DCM (12, 13). Previous studies have demonstrated that spinal cord compression revealed by MRI imaging correlates with the severity of neurological impairment and shows a predictive effect on the modified Japanese Orthopedic Association (mJOA) score 6 months post-surgery (14–18). However, spinal cord compression is presented in approximately 5% of asymptomatic individuals (19). Additionally, DCM usually involves a range of neurological impairments including motor weakness and sensory deficits, gait imbalance, and difficulties with urination and defecation (20). As one category of mJOA scores covers a wide range of clinical severity, the correlation between the sum of mJOA score and MRI parameters lacks specificity and persuasiveness (21). This highlights the insufficient understanding of the relationship between cervical cord compression and the variable clinical symptoms of DCM. Upper extremity impairment is one of the most typical neurological symptoms in DCM and causes substantial physical and social disability in daily life and seriously impacts patients' satisfaction (22, 23). To our knowledge, the relationship between upper extremity dysfunction and compressed cervical cord in DCM is still understudied.

In this study, we aimed to investigate whether the degree of compressed cervical cord is related to the severity of upper extremity symptoms in patients with DCM, and whether the degree of cervical cord compression in DCM patients can predict the presence of upper extremity motor impairment.

## Materials and methods

### Ethics

Our study protocol was approved by the Ethics Committee of the First Affiliated Hospital of Anhui Medical University (No. SL-YX2018-324) and was conducted in accordance with the Declaration of Helsinki. Informed consent was obtained from all individual participants included in the study. The authors affirm that human research participants provided informed consent for publication of the images.

### Study participants

**Figure 1:**
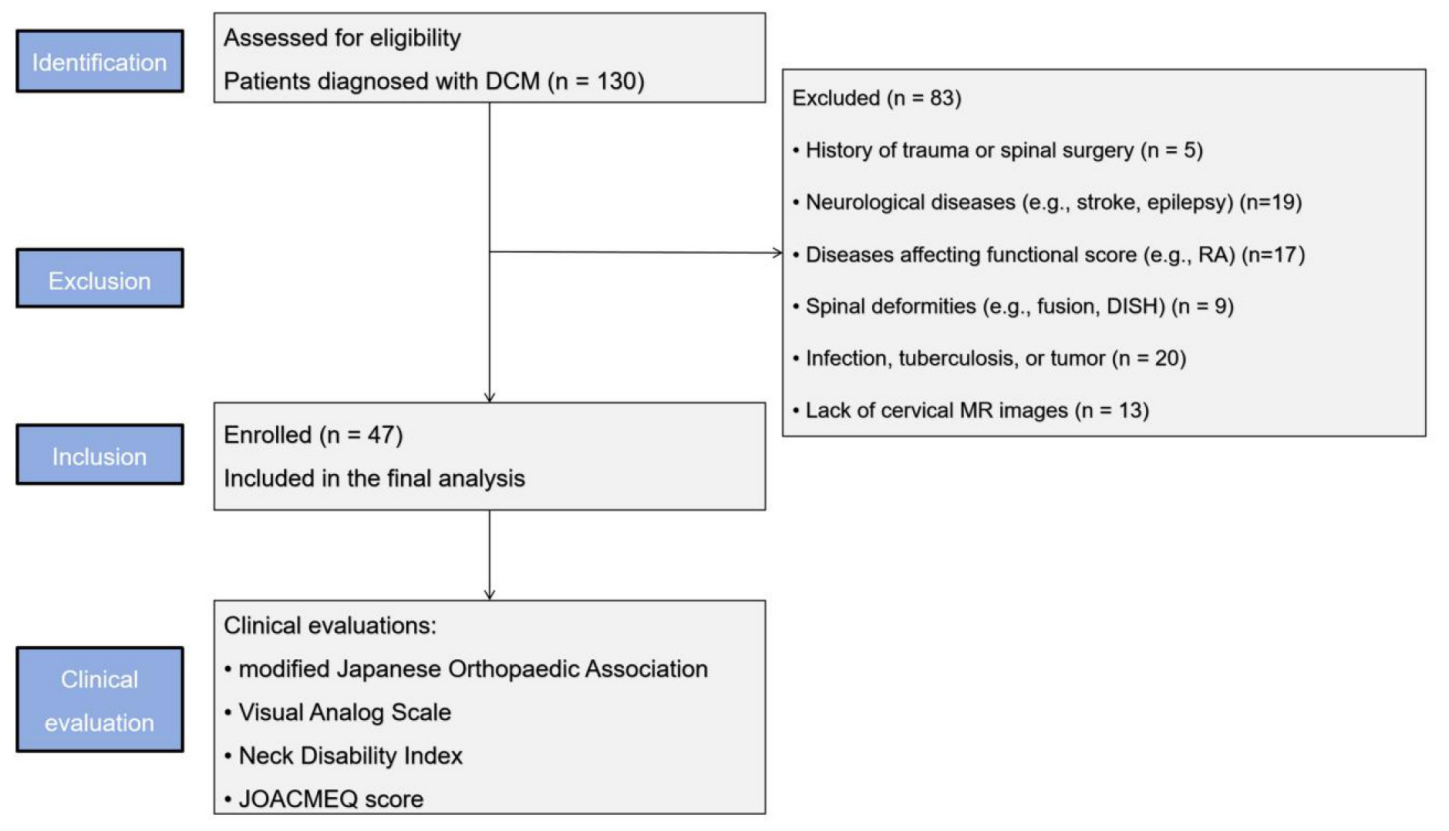
STROBE flow diagram of the study.

This study retrospectively analyzed data from 130 patients with DCM collected from January 2023 to May 2025 in the First Affiliated Hospital of Anhui Medical University. The inclusion criteria were as follows: (1) patients diagnosed with DCM based on their clinical symptoms including upper extremity weakness or clumsiness, gait disturbance, sensory deficits, and difficulties with urination and defecation, alongside imaging data including cervical X-ray, CT, and MRI; (2) patients demonstrated single-level of stenosis at C3/4-C6/7 level, defined as the obliteration of the subarachnoid space with spinal cord compression on T2-weighted MRI. The exclusion criteria were as follows: (1) a history of trauma or spinal surgery; (2) patients with other neurological diseases like stroke, Parkinson's disease, migraine and epilepsy; (3) patients with a condition that could affect the functional score, such as diabetes, ankylosing spondylitis, rheumatoid arthritis and other diseases; (4) the presence of congenital vertebral fusion, diffuse idiopathic skeletal hyperostosis, Klippel-Feil syndrome, cervical spondylolisthesis, or cervical instability; (5) the presence of an infection, tuberculosis, a tumor and another disease; (6) patients without cervical MR images. 47 patients were included in this study (Figure 1).

### Clinical examination

All patients were administered the mJOA scale, visual analog scale (VAS), neck disabled index (NDI) and the Japanese Orthopedic Association Cervical Myelopathy Evaluation Questionnaire (JOACMEQ) scores at the initial visit. (1) The mJOA scale consisted of four categories: upper extremity motor function, lower extremity motor function, upper extremity sensory function, and sphincter function. The total score was 18 points (24, 25). (2) VAS was a numeric rating scale ranging from zero (no pain) to 10 (worst pain imaginable). The VAS was defined as pain of the patient's neck and upper limbs, respectively (6, 26). (3) The NDI is a self-reported outcome measure consisting of 10 sections, with a total score of 100 points, used to evaluate the extent to which neck pain affects the patient's ability to manage everyday life. A higher score indicates more severe symptoms (27). (4) JOACMEQ consists five domains ranging from 0 to 100, these domains include cervical spine function, upper extremity function, lower extremity function, bladder function, and quality of life (24, 28).

The patients were divided into two groups according to the subsection of the mJOA score involving upper extremity motor function to serve as the reference standard for the ROC analysis: Group A (no upper extremity motor impairment, with an mJOA subscore for upper extremity motor function of five points) and Group B (with upper extremity motor impairment, mJOA subscore for upper extremity motor function of 4 points or less).

### MRI protocol

All patients underwent a 3.0T MRI scan (Siemens Medical Solutions, Erlangen, Germany) of the cervical spine by using a 20-channel head and neck matrix coil combined with a spine matrix coil. To precisely evaluate the characteristics of spinal cord, three dimensional T2 sampling perfection with application-optimized contrasts using different flip-angle evolutions (T2-SPACE) sequence was performed through the cervical spine in DCM patients (11). The T2-SPACE sequence involved the following imaging parameters: TE, 135 ms; TR, 1,500 ms; flip angle, 125 °; partition thickness, 0.4 mm; FOV, 250 × 250 mm and total acquisition time = 5:50 min.

### Image analysis

On T2-SPACE images, the spinal cord from C2 to C7 level were manually labeled and segmented using Jim 7.0 software by a 15-year experienced spine surgeon who was blinded to patients' information. The cross-sectional tissue areas (CSA), anterior-posterior width (APW), right-left width (RLW), the compression ratio (CR, CR = APW/RLW × 100%) of each slice were quantified by spinal cord toolbox (29). The presence of a hyperintense signal within the spinal cord parenchyma was defined as increased signal intensity on high-resolution 3D T2-SPACE images. Once observed, the length of the increased signal intensity (LISI) on sagittal view was measured (16). The most compressed slice of cervical cord in each DCM patient was recorded for analysis.

### Statistical analysis

SPSS 24.0 was used for statistical analyses. The normality of continuous variables was assessed using the Shapiro-Wilk test. Continuous variables conforming to a normal distribution were expressed as mean ± standard deviation (SD) and compared using the independent samples *t*-test. Variables that were not normally distributed were expressed as median (interquartile range, IQR) and compared using the Mann-Whitney *U*-test. Spearman correlation analysis was conducted to assess the relationships between the characteristics of the compressed cervical cord and upper extremity functional scores. To control for the false discovery rate arising from multiple comparisons, the Benjamini-Hochberg procedure was applied to adjust the significance levels for the correlation analyses. The strength of the correlation was interpreted based on the absolute value of the Spearman correlation coefficient: |ρ| = 0.10–0.39 indicates a weak correlation, |ρ| = 0.40–0.69 indicates a moderate correlation, and |ρ| = 0.70–1.00 indicates a strong correlation. Then, data on demographic characteristics, characteristics of compressed cervical cord and clinical outcome scores were compared between group A and group B using two-sample *t*-test. Sex differences between groups were examined using the chi-squared test. To identify risk factors for upper extremity motor dysfunction according to the mJOA score, univariate binary logistic regression analysis was employed. The critical value for predicting upper extremity motor dysfunction was determined using the Receiver Operating Characteristic (ROC) curve. The area under the curve (AUC) was calculated using Youden's Index to quantify its predictive accuracy. The AUC was interpreted as follows: poor discrimination (AUC = 0.5–0.69), moderate discrimination (AUC = 0.70–0.79), good discrimination (AUC = 0.80–0.89), and excellent discrimination (AUC ≥ 0.90). The confidence interval was set to 95%, and a *P*-value of 0.05 or less was regarded as a significant difference.

## Results

### Demographic and clinical characteristics

In this study, we recruited 47 DCM patients (29 males, 18 females). The mean age of DCM patients was 57.0 ± 11.6 years (mean ± standard deviation) and the mean body mass index (BMI) of DCM patients was 23.9 ± 3.2 (Table 1). The distribution of DCM patients according to the cervical cord compression segment is as follows: 9 patients at the C3/4 level, 11 patients at the C4/5 level, 22 patients at the C5/6 level, and five patients at the C6/7 level (Supplementary Table S1).

**Table 1:** Demographic participants' data.

**Variables**	**Values**	**Range [min; max]**
Age (years)	57.0 ± 11.6	[34:79]
Sex (male/female)	29/18	
BMI	23.9 ± 3.2	[18.3:31.2]
mean mJOA score	13.5 (11.0, 15.0)	[9:17]
mJOA subscore for upper extremity motor function	4.0 (3.0, 5.0)	[2:5]
mJOA subscore for lower extremity motor function	5.0 (4.0, 6.0)	[3:7]
mJOA subscore for upper extremity sensory function	2.0 (1.0, 2.0)	[1:3]
mJOA subscore for bladder function	3.0 (2.0, 3.0)	[1:3]
Mean NDI score	55.4 ± 20.2	[20:100]
VAS score for neck	4.0 (2.25, 5.0)	[0:7]
VAS score for upper extremity	5.0 (4.0, 6.0)	[0:8]
JOACMEQ subsection for cervical spine function	85.0 (65.0, 100.0)	[45:100]
JOACMEQ subsection for upper extremity function	84.2 (65.9, 94.7)	[26.3:100]
JOACMEQ subsection for lower extremity function	50.0 (36.4, 72.7)	[9.1:100]
JOACMEQ subsection for bladder function	87.5 (75.0, 100.0)	[25:100]
JOACMEQ subsection for quality of life	41.5 ± 11.3	[16.7:67.7]

Normally distributed data are presented as mean ± standard deviation (SD). Non-normally distributed data are presented as median (interquartile range, IQR).

BMI, body mass index; mJOA, modified Japanese Orthopedic Association; NDI, neck disability index; VAS, visual analog scale; JOACMEQ, Japanese Orthopedic Association Cervical Myelopathy Evaluation Questionnaire.

All DCM participants underwent the specified clinical examinations. The median mJOA score was 13.5 (IQR 11.0, 15.0). The median mJOA subscores were as follows: 4.0 (IQR 3.0, 5.0) for upper extremity motor function, 5.0 (IQR 4.0, 6.0) for lower extremity motor function, 2.0 (IQR 1.0, 2.0) for upper extremity sensory function, and 3.0 (IQR 2.0, 3.0) for sphincter function. The mean NDI score was 55.4 ± 20.2. The median VAS scores were 4.0 (IQR 2.25, 5.0) for neck pain and 5.0 (IQR 4.0, 6.0) for upper extremity pain. The median JOACMEQ subscores were 85.0 (IQR 65.0, 100.0) for cervical spine function, 84.2 (IQR 65.9, 94.7) for upper extremity function, 50.0 (IQR 36.4, 72.7) for lower extremity function, 87.5 (IQR 75.0, 100.0) for bladder function, and 41.5 ± 11.3 for quality of life in DCM patients (Table 1).

### Correlations between imaging parameters and functional scores

**Figure 2:**
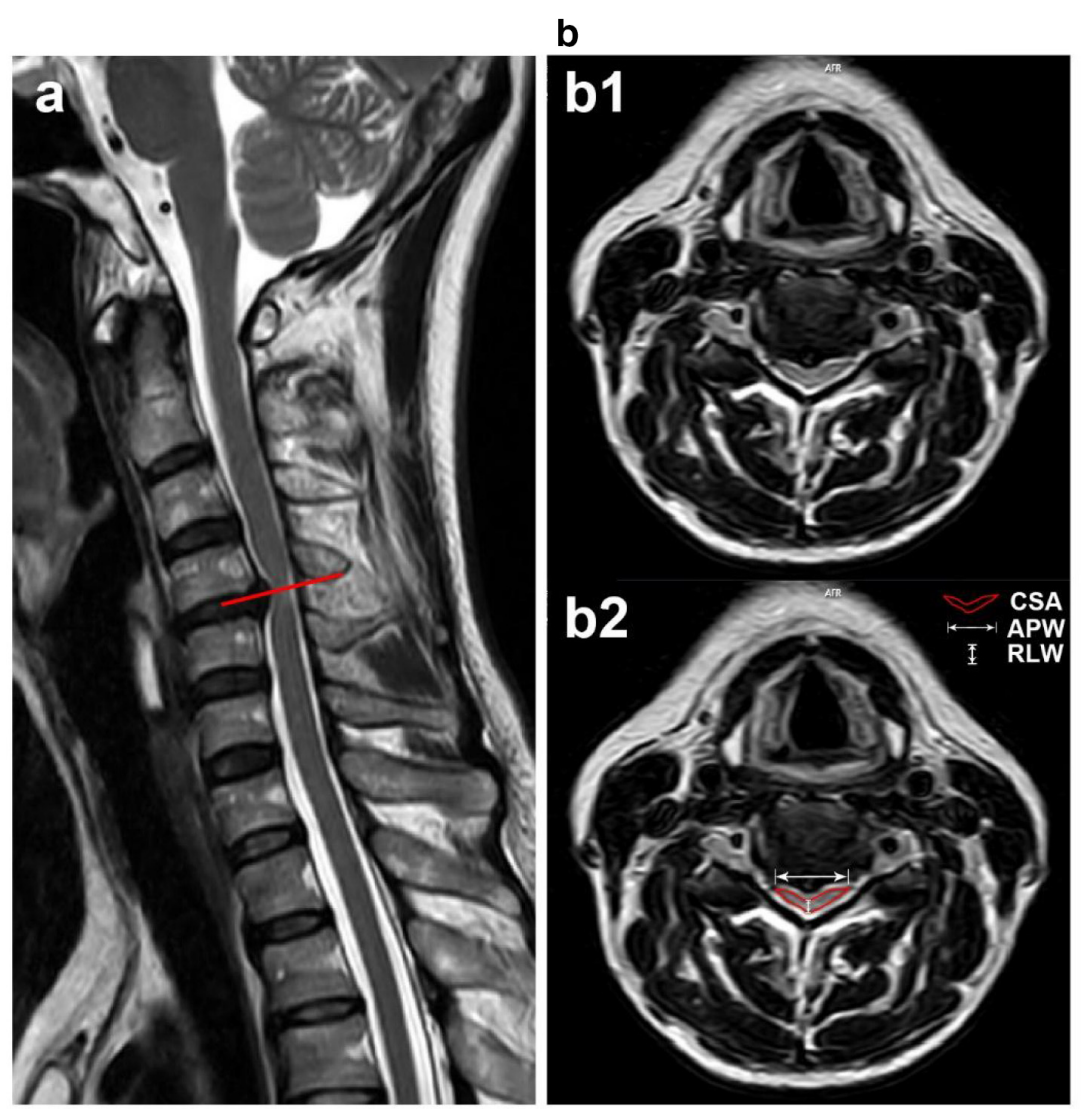
A T2-weighted MRI depicting a 53-year-old female with confirmed DCM. She complained of hand clumsiness and limb numbness for 6 months, with a total mJOA score of 14. Characteristics of the compressed cervical cord are shown: **(a)** Sagittal view showing C4/5 stenosis (indicated by the red line). **(b)** Transverse view (b1) showing the slice with the most severe cervical cord compression, with CSA, APW, and RLW marked in (b2).

The characteristics of the compressed cervical cord are illustrated in Figure 2. The mean CSA of the most compressed cervical cord slice in DCM patients was 42.4 ± 11.5 mm^2^. The mean APW of this slice was 3.9 ± 0.9 mm, the mean RLW was 14.8 ± 1.4 mm, and the mean CR was 26.5 ± 5.9%. Twenty-one patients (44.7%) presented with intramedullary hyperintensity with a mean LISI of 7.2 ± 10.7 mm (Supplementary Table S2), while 26 patients (55.3%) showed no evidence of intramedullary signal changes.

**Figure 3:**
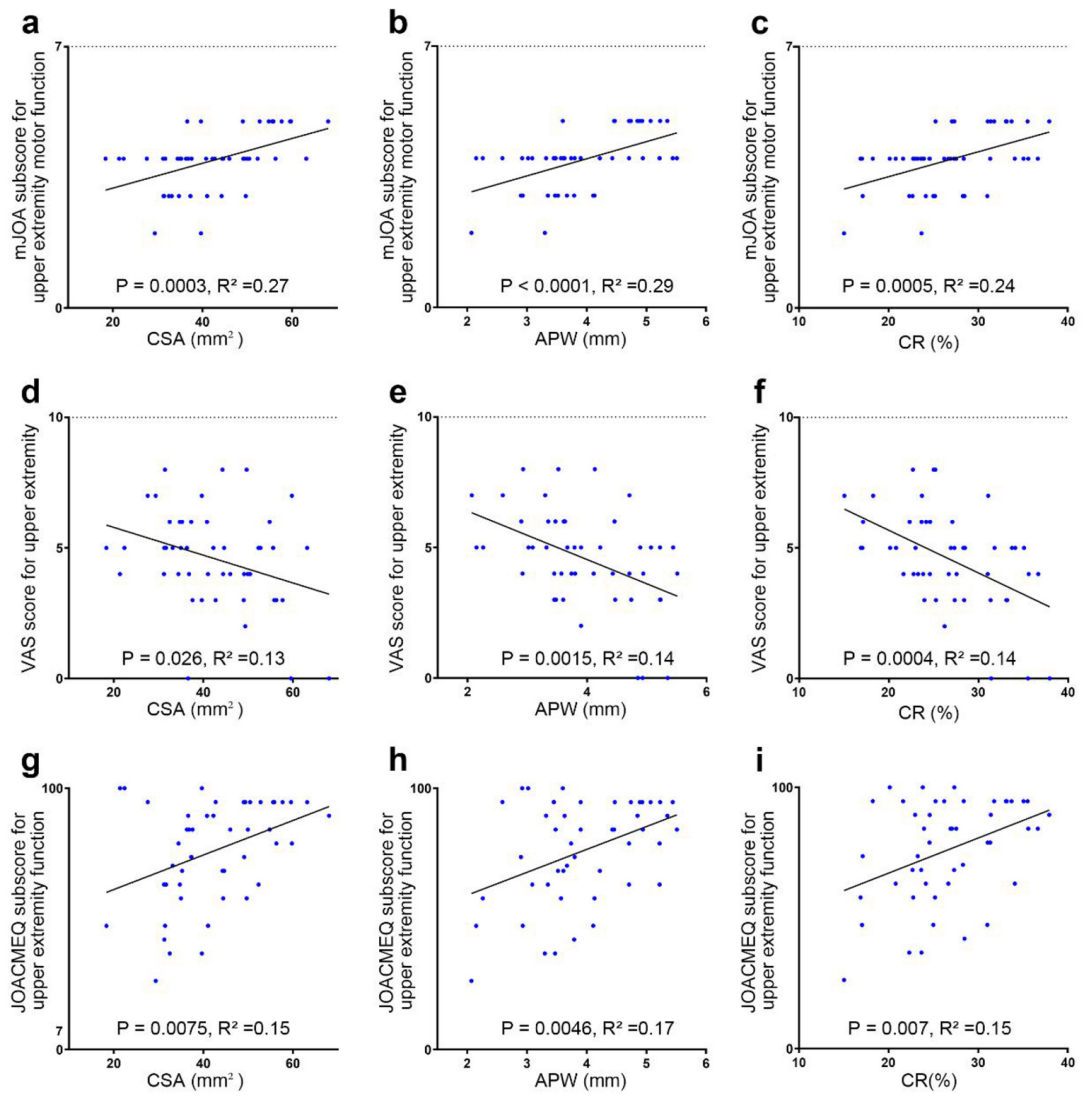
Relationship between characteristics of the compressed cervical cord and clinical outcomes. **(a–c)** Relationship between CSA, APW, and CR of the compressed cervical cord and the mJOA subscore for upper extremity motor function. **(d–f)** Relationship between CSA, APW, and CR of the compressed cervical cord and the VAS score for upper extremity function. **(g–i)** Relationship between CSA, APW, and CR of the compressed cervical cord and the JOACMEQ subscore for upper extremity function.

The CSA of the most compressed cervical cord in DCM patients was correlated with the mJOA score (ρ = 0.53, *P* < 0.01), the mJOA subscore for upper extremity motor function (ρ = 0.54, *P* < 0.01, Figure 3a, Table 2), the VAS score for upper extremity (ρ = −0.32, *P* < 0.05, Figure 3d, Table 2), and the JOACMEQ subscores for cervical spine function (ρ = 0.36, *P* < 0.05, Figure 3g, Table 2) and upper extremity function (ρ = 0.33, *P* < 0.05, Table 2). However, CSA was not correlated with mJOA subscore for upper extremity sensory function and bladder function, mean NDI score, VAS score for neck, JOACMEQ subsection for lower extremity function, bladder function and quality of life in DCM patients (Table 2).

**Table 2:** Correlation between clinical evaluations and the characteristics of the compressed cervical cord.

**Variables**	**CSA**	**APW**	**RLW**	**CR**	**LISI**
mJOA score	0.53^**^	0.37^*^	0.00	0.42^**^	−0.16
mJOA subscore for upper extremity motor function	0.54^**^	0.54^**^	0.20	0.48^**^	−0.19
mJOA subscore for lower extremity motor function	0.33^*^	0.08	−0.12	0.19	−0.20
mJOA subscore for upper extremity sensory function	0.17	0.30^*^	0.02	0.31^*^	0.06
mJOA subscore for bladder function	0.13	0.07	−0.06	0.12	0.00
Mean NDI score	−0.26	0.23	−0.15	−0.20	0.08
VAS score for neck	−0.17	−0.20	−0.02	−0.25	0.01
VAS score for upper extremity	−0.32^*^	−0.44^**^	−0.02	−0.46^*^	0.01
JOACMEQ subsection for cervical spine function	0.36^*^	0.30^*^	0.09	0.35^*^	0.00
JOACMEQ subsection for upper extremity function	0.33^*^	0.30^*^	0.03	0.33^*^	−0.07
JOACMEQ subsection for lower extremity function	0.21	0.05	0.00	0.13	−0.24
JOACMEQ subsection for bladder function	0.27	0.27	−0.08	0.35^*^	0.01
JOACMEQ subsection for quality of life	0.07	0.06	−0.12	0.17	0.07

mJOA, modified Japanese Orthopedic Association; NDI, neck disability index; VAS, visual analog scale; JOACMEQ, Japanese Orthopedic Association Cervical Myelopathy Evaluation Questionnaire; CSA, cross-sectional tissue areas; APW, anterior-posterior width; RLW, right-left width; CR, compression ratio; LISI, length of increased signal intensity.

Spearman correlation coefficients are shown. ^*^P < 0.05, ^**^P < 0.01.

The APW of the most compressed cervical cord in DCM patients was correlated with the mJOA score (ρ = 0.37, *P* < 0.05, Table 2), the mJOA subscores for upper extremity motor function (ρ = 0.54, *P* < 0.01, Figure 3b, Table 2) and upper extremity sensory function (ρ = 0.30, *P* < 0.05, Table 2), the VAS score for upper extremity (ρ = −0.44, *P* < 0.01, Figure 3e, Table 2), and the JOACMEQ subscores for cervical spine function (ρ = 0.30, *P* < 0.05, Table 2) and upper extremity function (ρ = 0.30, *P* < 0.05, Figure 3h, Table 2). However, APW was not correlated with mJOA subscore for lower extremity sensory function and bladder function, mean NDI score, VAS score for neck, JOACMEQ subsection for lower extremity function, bladder function and quality of life in DCM patients (Table 2).

The CR of the most compressed cervical cord in DCM patients was correlated with mJOA score (ρ = 0.42, *P* < 0.01, Table 2), the mJOA subscore for upper extremity motor function (ρ = 0.48, *P* < 0.01, Table 2, Figure 3c) and upper extremity sensory function (ρ = 0.31, *P* < 0.05, Table 2), the VAS score for upper extremity (ρ = −0.46, *P* < 0.01, Figure 3f, Table 2), and the JOACMEQ subscores for cervical spine function (ρ = 0.35, *P* < 0.05, Table 2), for upper extremity function (ρ = 0.33, *P* < 0.05, Figure 3i, Table 2) and for bladder function (ρ = 0.35, *P* < 0.05, Table 2). In fact, CR was not correlated with mJOA subscore for lower extremity sensory function and bladder function, mean NDI score, VAS score for neck, JOACMEQ subsection for lower extremity function and quality of life in DCM patients (Table 2).

RLW and the LISI on T2 weighted imaging were not correlated with clinical outcome (Table 2).

### ROC curve to determine the critical values

**Figure 4:**
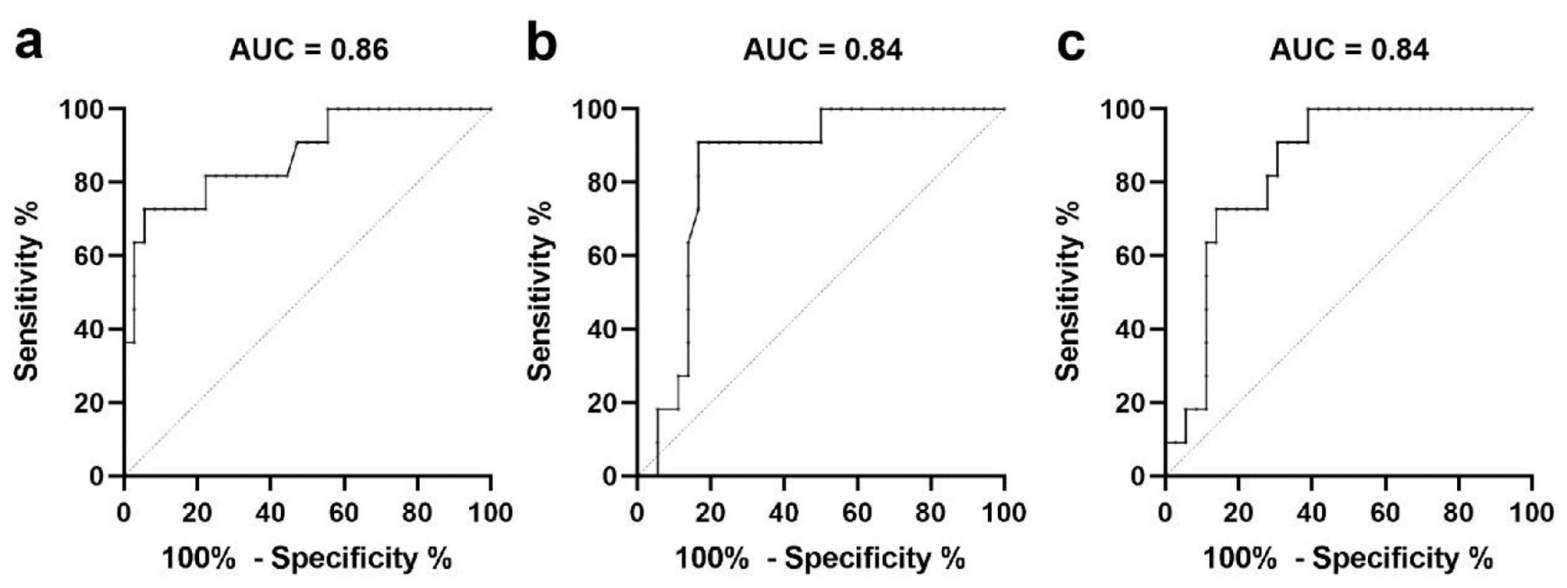
ROC curve showed discriminative ability of **(a–c)** CSA, APW, and CR for the mJOA subscore for upper extremity motor dysfunction in DCM participants.

DCM patients were divided into two groups according to upper extremity motor function of mJOA score. There were no significant differences in age, sex, BMI, mJOA subscore for lower extremity motor function, upper extremity sensory function and bladder function, mean NDI score, mean VAS score for neck pain, JOACMEQ subsection for cervical spine function, lower extremity function, bladder function, and quality of life between Group A (*n* = 12) and Group B (*n* = 35; Supplementary Table S3). However, the mean mJOA score, VAS score for upper extremity pain, JOACMEQ scores for upper extremity function, CSA, APW and CR were significantly different between the groups (*P* < 0.05, Supplementary Table S3). Binary logistic regression analysis found that CSA, APW, and CR were significant risk factors for upper extremity motor dysfunction as measured by the mJOA score (*P* < 0.01, Supplementary Table S4). ROC curve analysis revealed that DCM patients may demonstrate upper extremity motor dysfunction when CSA is less than 52.6 mm^2^ (*P* < 0.001, AUC = 0.86, sensitivity = 75.0%, specificity = 80.0%, Youden index = 0.55; Figure 4a, Table 3), APW is less than 4.5 mm (*P* < 0.001, AUC = 0.84, sensitivity = 75.0%, specificity = 82.9%, Youden index = 0.58; Figure 4b, Table 3), or CR is less than 31.4% (*P* < 0.001, AUC = 0.84, sensitivity = 66.7%, specificity = 82.9%, Youden index = 0.50; Figure 4c, Table 3).

**Table 3:** The discriminative ability of CSA, APW, and CR for upper extremity motor function of mJOA score in DCM participants.

**Parameter**	**Area**	**Standard deviation**	***P*-value**	**Cutoff value**	**Sensitivity**	**Specificity**	**Youden index**
CSA (mm^2^)	0.86	0.06	<0.001^***^	52.6	75.0%	80.0%	0.55
APW (mm)	0.84	0.06	<0.001^***^	4.5	75.0%	82.9%	0.58
CR (%)	0.84	0.06	<0.001^***^	31.4	66.7%	82.9%	0.50

CSA, cross-sectional tissue areas; APW, anterior-posterior width; RLW, right-left width; CR, compression ratio.

^***^P <0.001.

## Discussion

This study demonstrated the involvement of cervical cord compression in upper extremity function and pain but not gait, bladder, and sensory dysfunction following DCM. Our study also identified the characteristics of the cervical cord compression as predictors of upper extremity motor dysfunction. These findings allow us to critically discuss the value of cervical cord compression in diagnostic assessments and treatment planning for DCM.

The pathophysiology changes of DCM involve inflammation, ischemia, and apoptosis of neurons and oligodendrocytes triggered by chronic spinal cord compression (10). Previous studies have confirmed that CSA and CR are closely correlated with the degree of demyelination, axon degeneration, and neuronal necrosis changes in DCM (12). In 1988, Keiro first introduced CSA and CR to capture the size and shape of the compressed cervical cord. When the CR is below 20%, severe compression leads to extensive demyelination of the lateral funiculus, and further compression can result in necrosis of the central gray matter (14, 15). These pathologial changes contribute to a notable decline in clinical evaluations. In our cross-sectional study, we found that the more severe the spinal cord compression, the worse the mJOA score. Moreover, previous cohort studies have indicated that MRI parameters, as assessed by CSA of cervical cord are strong predictors of postsurgical mJOA score at 6 months in DCM patients (17). Therefore, the characteristics of the compressed cervical cord are closely associated with baseline neurological impairment and postsurgical recovery, emphasizing their critical role in the management and prognosis of DCM.

Upper extremity dysfunction is prevalent in DCM patients and significantly contributes to clinical disability (20). While sensory impairment is reportedly more prevalent than motor impairment, especially in mild cases, upper extremity motor function is considered more critical for daily activities and functional independence (26). DCM patients with less improvement in their upper extremity motor score and bodily pain were more likely to be dissatisfied with their outcomes following surgery (27). To assess upper limb function, we enrolled 47 single-level DCM patients, conducted a detailed classification of the mJOA, VAS, and JOACMEQ scores and recorded the subscore of upper extremity function. Here, we found that the extent of cervical cord compression in DCM patients is correlated with subsections of the mJOA score, VAS score, and JOACMEQ score involving the upper extremity function. According to human post-mortem studies and animal models of DCM, chronic cervical cord compression may trigger Fas/Fas ligand-mediated apoptosis of neurons and oligodendrocytes, along with inflammation, which impacts neuronal function and survival in DCM (30). These pathological changes are characterized by demyelination, axonal loss in the posterior and lateral funiculi, and necrosis with cavity formation in the gray matter (31). Alterations in motor neurons, sensory neurons in the gray matter and spinothalamic tract in the lateral funiculus may result in motor weakness, finger spasticity, and pain (32). In brief, our study is the first to explore the correlation between the compression of the cervical cord and upper extremity function in DCM, providing evidence and explanations that support previous research while also deepening the understanding of neurological function in DCM.

In fact, the spinal cord is relatively resistant to compression. A cross-sectional population study of 1,211 asymptomatic participants found that 5.3% of subjects had evidence of spinal cord compression from degenerative changes without clinical symptoms (33). In fact, cervical cord compression evaluated by MRI measurements alone may not fully predict functional impairment. Golash et al. (34) reported that CSA can be an insensitive indicator of myelopathy, with significant overlap observed between patients with and without clinical symptoms. As noted in reviews by Nouri et al. (35) and Akter and Kotter (36), the pathobiology of DCM involves complex mechanisms beyond mechanical constriction—such as ischemia, blood-spinal cord barrier disruption, and neuroinflammation—which do not always linearly correspond to the degree of compression. Previous studies have also reported that the spinal cord loses its functional tolerance if the transverse area, as measured by computed tomographic myelography and MRI, is 55%−75% of the normal value (37, 38). In another study, the critical value for intolerance to compression for spinal cord function was found between 50 and 60 mm^2^ in 243 DCM patients (13). To identify the clinically significant degree of compression, which was associated with corresponding upper extremity motor dysfunction, we enrolled DCM patients with single-level stenosis to investigate the critical value of cervical cord compression. Our finding showed when CSA is less than 52.6 mm^2^, or APW is less than 4.5 mm or CR is less than 31.4%, the DCM patient may demonstrate upper extremity motor impairment in mJOA subscore. However, the various parts of the spinal cord differ in their sensitivity to external stress, as the gray matter of the cord is softer and more fragile and often an area of higher stress of distribution and lower blood perfusion than the white matter (36, 39–41). Another clinicopathologic study uncovered the common progression pattern of DCM: atrophy and neuronal loss in the anterior horn and intermediate zone develop first, followed by degeneration of the lateral and posterior funiculi (42, 43). In human DCM studies, significant decline in CSA of gray matter and spinal cord has been observed at the level of stenosis (44). While histopathological findings in human cases demonstrated significant atrophy of the anterior horn with marked neuronal loss and axonal swellings in the corticospinal tracts the compressed epicenter when compared with control cases (30). The motor neurons are located within the ventral horn in gray matter, innervating the extremity muscles (45). The observed degeneration in the central area and lateral columns of the corresponding segments might explain the upper extremity motor dysfunctions in DCM participants.

Our research has several limitations. First, the sample size was relatively small due to strict inclusion criteria enrolling only single-level DCM patients in single center. This approach tends in include DCM patients with soft disc herniations, may have introduced selection bias and does not consider the situation of patients with multi-level cord compression. Consequently, although we identified significant predictors, the strength of the correlations and the discriminative ability in the ROC analysis were moderate. In the future, large-scale studies are needed to validate these findings. Second, this study was a retrospective cross-sectional analysis focused on correlations between MRI and clinical evaluations. Patients receiving conservative treatment were excluded from the study cohort as they typically present with milder symptoms and often lack the systematic objective clinical data required for this analysis. Third, the neurological assessment in this study primarily relied on the mJOA score, which provides a relatively subjective and coarse evaluation of functional status. As a scoring system, it has limited sensitivity for detecting subtle changes and cannot precisely localize specific spinal cord involvement. In the future, our study will complement neurophysiological evaluations and incorporate both preoperative and postoperative measurements to better predict the relationship between cervical spinal cord compression and postsurgical recovery. Fourth, advanced quantitative microstructural MRI protocols, which are highly sensitive to the progression of myelopathy, were not applied in this study (46). In the future, DTI metrics, magnetization transfer, MR spectroscopy, and T2^*^-weighted imaging will be applied to investigate injury in patients with asymptomatic cervical spinal cord compression (10). Finally, CSA, APW, and CR possess a high degree of collinearity, which could imply redundancy in clinical application. However, our ROC analysis revealed that CSA demonstrated the superior discriminative ability compared to APW and CR, thus we recommend CSA as the most robust single biomarker for precise diagnostic assessment.

## Conclusion

In brief, the characteristics of compressed cervical cord are related to the upper extremity motor dysfunction and pain of DCM patients. This correlation not only deepens the understanding of neurological function but also provides an objective indicator for clinical assessment, valuable for refining clinical trial subgroups, guiding neurological prognostication and surgical decision-making processes. In the future, this clinical utility warrants further validation in larger, multicenter, and longitudinal studies.

## Data Availability

The raw data supporting the conclusions of this article will be made available by the authors, without undue reservation.
