# Supplementary material for: Association between quantitative cervical cord compression metrics and upper extremity impairments in degenerative cervical myelopathy: a cross-sectional study
Source: Front Neurol. 2026 Feb 20;17:1728273. doi: 10.3389/fneur.2026.1728273 (PMC12962929; doi:10.3389/fneur.2026.1728273)
Supplement: Supplementary file 1 [file Table_1.doc]

**Table S1: The distribution of DCM patients according to the cervical cord compression segment**

| The compression level of the cervical cord | N (Percentage) |
| --- | --- |
| C3/4 | 9 (19) |
| C4/5 | 11 (23) |
| C5/6 | 22 (46) |
| C6/7 | 5 (11) |
