# Supplementary material for: Association between quantitative cervical cord compression metrics and upper extremity impairments in degenerative cervical myelopathy: a cross-sectional study
Source: Front Neurol. 2026 Feb 20;17:1728273. doi: 10.3389/fneur.2026.1728273 (PMC12962929; doi:10.3389/fneur.2026.1728273)
Supplement: Supplementary file 2 [file Table_2.doc]

**Table S2: MRI parameters of the compressed cervical cord**

| Variable | Values | Range [min; max] |
| --- | --- | --- |
| CSA (mm²) | 42.4 ± 11.5 | [21.4: 68.1] |
| APW (mm) | 3.9 ± 0.9 | [2.1: 5.5] |
| RLW (mm) | 14.8 ± 1.4 | [11.7:17.7] |
| CR (%) | 26.5 ± 5.9 | [15.0: 37.9] |
| LISI (mm) | 7.2 ± 10.7 | [0: 56.5] |

Values are presented as or mean±standard deviation.

CSA, cross-sectional tissue areas; APW, anterior-posterior width, RLW, right-left width; CR, compression ratio; LISI, length of increased signal intensity
