# Supplementary material for: Association between quantitative cervical cord compression metrics and upper extremity impairments in degenerative cervical myelopathy: a cross-sectional study
Source: Front Neurol. 2026 Feb 20;17:1728273. doi: 10.3389/fneur.2026.1728273 (PMC12962929; doi:10.3389/fneur.2026.1728273)
Supplement: Supplementary file 3 [file Table_3.doc]

**Table V: Basic data of Group A (n=12) and Group B (n=35)**

|  | Group A (No upper extremity motor dysfunction, n = 12) | | Group B (With upper extremity motor dysfunction, n = 35) | |  |
| --- | --- | --- | --- | --- | --- |
| Parameter | Values | Range [min; max] | Values | Range [min; max] | P value |
| Age (years) | 58.1 ± 11.6 | [42: 79] | 56.7 ± 11.7 | [34: 79] | 0.73 |
| Sex (Male/Female) | 8/3 | - | 21/15 | - | 0.4 |
| BMI | 24.9 ± 2.4 | [21.0: 28.4] | 23.6 ± 3.4 | [18.3: 31.2] | 0.25 |
| mean mJOA score | 14.8 ± 1.4 | [13: 17] | 12.6 ± 1.9 | [9: 16] | <0.001*** |
| mJOA subscore for lower extremity motor function | 5.3 ± 1.3 | [3: 7] | 4.7 ± 1.1 | [3: 7] | 0.17 |
| mJOA subscore for upper extremity sensory function | 2.0 ± 0.6 | [1: 3] | 1.8± 0.6 | [1: 3] | 0.24 |
| mJOA subscore for bladder function | 2.6 ± 0.70 | [1: 3] | 2.5 ± 0.5 | [2: 3] | 0.93 |
| mean NDI score | 47.2 ± 17.6 | [28: 88] | 57.8 ± 20.6 | [20: 100] | 0.13 |
| VAS score for neck | 3.6 ± 2.3 | [0: 7] | 3.6 ± 1.8 | [0: 6] | 0.99 |
| VAS score for upper extremity | 3.2 ± 2.4 | [0: 7] | 5.0 ± 1.5 | [2: 8] | < 0.01** |
| JOACMEQ subsection for cervical spine function | 90.0 ± 11.8 | [65: 100] | 75.8 ± 19.3 | [45: 100] | 0.09 |
| JOACMEQ subsection for upper extremity function | 91.9 ± 5.9 | [78.9: 100.0] | 71.3 ± 19.9 | [26.3: 100.0] | < 0.01** |
| JOACMEQ subsection for lower extremity function | 66.1 ± 24.6 | [22.7: 100.0] | 52.9 ± 24.4 | [9.09 - 100.0] | 0.12 |
| JOACMEQ subsection for bladder function | 87.5 ± 14.5 | [56.3: 100.0] | 82.0 ± 16.5 | [25.0: 100.0] | 0.32 |
| JOACMEQ subsection for quality of life | 44.9 ± 14.5 | [27.1: 67.7] | 40.6 ± 10.4 | [16.7: 57.3] | 0.29 |
| CSA (mm²) | 53.6 ± 9.0 | [36.6: 68.1] | 38.7 ± 9.3 | [21.4: 51.3] | <0.001*** |
| APW (mm) | 4.8 ± 0.5 | [3.6: 5.4] | 3.7 ± 0.9 | [2.1: 5.5] | <0.001*** |
| RLW (mm) | 15.1 ± 1.3 | [13.2: 17.7] | 14.7 ± 1.4 | [11.7: 17.7] | 0.35 |
| CR (%) | 31.6 ± 3.8 | [25.2: 37.9] | 25.1 ± 5.3 | [15.0: 36.7] | <0.001*** |
| LISI (mm) | 5.1 ± 8.4 | [0: 25.8] | 7.9 ± 11.3 | [0: 56.5] | 0.39 |

Note: Values are presented as mean±standard deviation.

BMI, body mass index; mJOA, modified Japanese Orthopaedic Association; NDI, neck disability index; VAS, visual analog scale; JOACMEQ, Japanese Orthopaedic Association Cervical Myelopathy Evaluation Questionnaire, CSA, cross-sectional tissue areas; APW, anterior-posterior width, RLW, right-left width; CR, compression ratio; LISI, length of increased signal intensity. ** P < 0.01, *** P < 0.001.
