# Supplementary material for: Association between quantitative cervical cord compression metrics and upper extremity impairments in degenerative cervical myelopathy: a cross-sectional study
Source: Front Neurol. 2026 Feb 20;17:1728273. doi: 10.3389/fneur.2026.1728273 (PMC12962929; doi:10.3389/fneur.2026.1728273)
Supplement: Supplementary file 4 [file Table_4.doc]

**Table S4: Binary logistic regression analysis to identify risk factors for upper extremity motor function of mJOA score in DCM participants.**

|  | Coefficient B | P value | Odds Ratio | 95% Confidence Interval of Odds Ratio | |
| --- | --- | --- | --- | --- | --- |
|  |  |  |  | Lower bound | Upper bound |
| CSA | 0.19 | < 0.01** | 1.21 | 1.07 | 1.37 |
| APW | 1.77 | < 0.01** | 5.88 | 1.85 | 18.7 |
| RLW | 0.24 | 0.35 | 1.28 | 0.76 | 2.13 |
| CR | 0.25 | < 0.01*** | 1.29 | 1.09 | 1.52 |
| LISI | -0.03 | 0.46 | 0.97 | 0.89 | 1.05 |

Note: CSA, cross-sectional tissue areas; APW, anterior-posterior width, RLW, right-left width; CR, compression ratio; LISI, length of increased signal intensity; mJOA, modified Japanese Orthopaedic Association; DCM, degenerative cervical myelopathy. ** P < 0.01, *** P < 0.001.
